# A GIS-Based Approach for Flood Risk Zoning by Combining Social Vulnerability and Flood Susceptibility: A Case Study of Nanjing, China

**DOI:** 10.3390/ijerph182111597

**Published:** 2021-11-04

**Authors:** Yi Chen, Zhicong Ye, Hui Liu, Ruishan Chen, Zhenhuan Liu, Hui Liu

**Affiliations:** 1 School of Architecture, Nanjing Tech University, Nanjing 211816, China; chenyi2012@njtech.edu.cn (Y.C.); yezhicong@njtech.edu.cn (Z.Y.); louis_liu@njtech.edu.cn (H.L.); 2 School of Government, Central University of Finance and Economics, Beijing 100081, China; 3 School of Design, Shanghai Jiao Tong University, Shanghai 200240, China; rschen@geo.ecnu.edu.cn; 4 School of Geography and Planning, Sun Yat-sen University, Guangzhou 510275, China; liuzhh39@mail.sysu.edu.cn

**Keywords:** social vulnerability, flood susceptibility, risk zoning, community, Nanjing

## Abstract

The identification of vulnerable people and places to flood is crucial for effective disaster risk management. Here, we combine flood hazard and social vulnerability index to capture the potential risk of flood. In this paper, Nanjing was taken as the case study to explore the spatial pattern of social vulnerability towards flood at the community scale by developing an index system. Based on the flood risk results of ArcSWAT, the risk of flood disaster in Nanjing was evaluated. The results show the following. (1) Social vulnerability exhibits a central–peripheral pattern in general, which means that the social vulnerability degree is high in the central city and decreases gradually to the suburbs. (2) The susceptibility to flood disaster has a similar circle-layer pattern that is the highest in the urban centre, lower in the exurban areas, and the lowest in the suburb areas. (3) By using the GIS-based zoning approach, communities are classified into four types by comprehensively considering their flood susceptibility and social vulnerability. The spatial pattern is explained, and policy recommendation for reducing flood risk is provided for each type of community. The research has important reference significance for identifying the spatial pattern of social vulnerability to flood and then formulating targeted adaptation countermeasures.

## 1. Introduction

Flood disasters are expected to become more common and devastating than before in the most densely inhabited urban areas [1]. Flood susceptibility has increased in built-up areas as a result of rising population and asset consolidation [2]. Therefore, solutions for adapting to urban floods must be developed, particularly for those who are most susceptible in flood-prone areas.

The most frequent technique used for coping with the potential consequences of flooding disasters is flood risk management. Traditionally, flood risk assessments have considered physical vulnerability but have mainly overlooked social vulnerability [3,4]. Household adaptability is critical for risk-reduction policies, including individual risk mitigation and evacuation planning [4]. To estimate the danger of urban flooding, these areas can be integrated with social vulnerability and flood susceptibility to reflect the potential risk.

Social vulnerability to flood has been evaluated in different spatial scales, including national, regional, and local [5,6,7,8]. Most of these studies evaluated social–economic variables to explore the level of social vulnerability, while the spatial pattern of social vulnerability was rarely addressed from a central–peripheral perspective. On the one hand, migrants, females, the elderly, children, and public services are all closely linked to urban structure. On the other hand, the theory of social differentiation can be well understood through the spatial pattern of SoVI. The goal of this study is three-fold. Firstly, the current study contributes to the literature by analysing the social vulnerability to flood in China, emphasising the need for a social vulnerability index (SoVI) assessment at the community scale. Secondly, a comprehensive indicator system is developed for assessing social vulnerability to flood in rapidly urbanising countries, which have low-density expansion and high-density infill simultaneously. Thirdly, a GIS-based zoning method is developed to classify communities into different types by simultaneously considering their flood susceptibility and social vulnerability.

This paper is divided into four parts. Firstly, taking Nanjing as the research area and its neighbourhoods as the statistical unit, the social vulnerability evaluation index is constructed according to the characteristics of the case area, and the social vulnerability map of Nanjing is obtained. Secondly, the flood disaster susceptibility map of Nanjing is obtained by analysing its flood disaster susceptibility through hydrological simulation. Thirdly, social vulnerability and flood disaster susceptibility are combined to explore the spatial pattern characteristics of flood disaster risk in Nanjing. Finally, the research findings and conclusions are presented.

## 2. Social Vulnerability and Flood Susceptibility

Social vulnerability is a product of social inequalities; despite the fact that social vulnerability is feasible [9], the SoVI assessment approach is still widely used [10]. SoVI has been effectively implemented in various applications and spatial dimensions [5,6,7,8,11]. In disasters, social vulnerability can be used to identify vulnerable groups [10,12,13]. The importance of society and human individual factors as hazard determinants must be recognised [14,15]. In the context of natural disasters, social vulnerability is intertwined with vulnerable people or social issues, such as poverty.

Flood susceptibility assessment can aid emergency management in determining whether areas are at risk of flooding. Three basic domains exist in which flood susceptibility mapping approaches can be classified: hydrological models, such as SWAT; statistical approaches, such as fuzzy logic; and machine learning, such as random forest [16,17,18]. Traditional flood risk management assess losses of flood by water depth, neglecting the social dimensions of flood risk [19]. In comparison with traditional flood risk management, flood susceptibility has undergone a paradigm shift, which emphasises the integration of social factors into flood risk analysis [4,6,20,21].

The combination of social vulnerability and flood susceptibility is necessary to provide valuable information for flood risk management. The spatial heterogeneity of the population is often ignored in previous research; therefore, flood risk management should analyse the socioeconomic characteristics of individual households [4,20].

In China, Zeng et al. (2012) used high spatial resolution satellite images to assess social vulnerability at a county scale in Guangzhou’s Luogang District [22]. Zhang and Huang (2013) explored the analytic hierarchy process approach to assess the SoVI of subdistricts for public safety in Beijing [23]. Zhou, Li, Wu, Wu, and Shi (2014) assessed societal vulnerability to natural disasters from the influence of local spatial and temporal factors [24]. At regional scales, Huang, Su, and Zhang (2015) assessed SoVI to natural disasters in the Beijing–Tianjin–Hebei region [25]. Ge, Wen, and Dai (2017) explored a novel method for identifying SoVI to climate change in the Yangtze River Delta, China [26]. Gu et al. (2018) conducted community-level social vulnerability and risk management for 5,342 communities in Shanghai, China [27]. Song et al. (2019) found that 39% of Shenzhen subdistricts are vulnerable to urban floods, with the majority of them being in exurban and suburban areas [28]. Li et al. (2021) assessed the vulnerability of rainfall disasters in Liaoning Province, China by using the ESA conceptual framework [29].

## 3. Framework and Method

### 3.1. Framework

Key dimensions of vulnerability include three aspects: environmental, social, and economic [30]. Environmental dimension focuses on coupled human–environmental systems. Whilst society development enhances social resilience by creating wealth, it can undermine ecosystem resilience if out of control [31]. The social dimension includes issues such as poverty, social marginalisation, vulnerable age groups, education, health, and well-being [5,9]. The economic dimension can refer to the economic assets of households at risk on the one hand, and on the other hand, it can refer to the susceptibility of an economic system [9,32].

**Figure 1:**
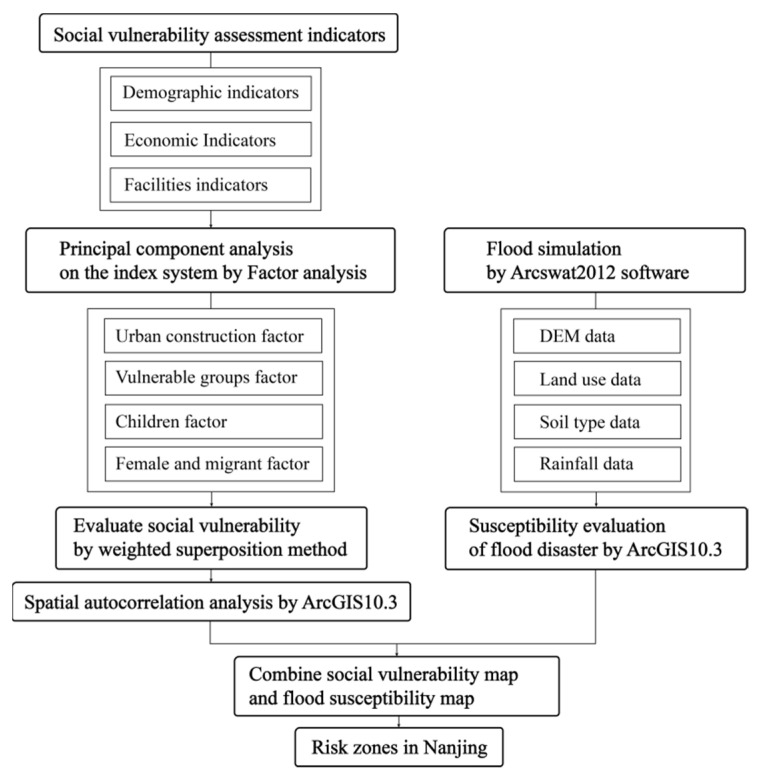
Flow chart for social vulnerability and flood susceptibility.

The interplay of hazardous physical occurrences with sensitive social conditions has increased the probability of catastrophe risk [30]. On the basis of relevant literature [5,15,17,27], we consider a combination of social vulnerability and flood susceptibility assessment for risk delineation. With the process of urbanisation, Nanjing is facing increasingly severe threats of rainstorm and flood [16]. Therefore, flood disaster is regarded as a main disturbance factor, and the social vulnerability assessment steps are as follows (Figure 1).

### 3.2. Variables

Based on a review of disaster and vulnerability literature [9,12,15,33], variables used in this analysis are presented in Table 1. Demographic variables may represent individuals’ or groups’ proclivity to be negatively impacted, such as physical limits, lack of access to services, and difficulties understanding and obtaining recovery information [5,9,12]; these variables are age structure (percentage of population ≥75 years, ≤14 years, under kindergarten and primary school age), education (percentage of population ≤ nine years of compulsory education, percentage of illiterate population), gender (percentage of females), rural (percentage of agricultural household registration), immigrant (percentage of immigrants), and population density (people per km^2^ of land area).

Economic indicators are composed of home value and construction density. Home value plays a positive role in measuring social status, obtaining resources, and attracting insurance from disaster and extreme events [12,15,34,35,36]. The average house price, housing area per capita, and retail density capture the characteristics of home value, all of which were used to reflect the main economic assets of households. Retail density was used to indicate the accessibility of living resources around settlement, such as water, food, appliances, and other commodities. The high density of construction increases the potential loss of physical assets and makes the agglomerated population have high vulnerability [5,9].

Facility indicators identifying the characteristics of service-oriented public facilities were collected from the points of interest (POIs) of maps. Insecure conditions are distinct manifestations of the vulnerability of a population in time and space in connection with a hazard [15]. When an infrastructure is unable to withstand extreme events, people using the infrastructure will be in unsafe conditions and have high social vulnerability [30,37]. The densities of kindergarten, primary school, and middle school were used to capture unsafe conditions arising from the education infrastructure. Meanwhile, the density of nursing homes was used to catch another unsafe condition caused by the agglomeration of elders. Service-oriented public facility indicators include the densities of bus stations, parks, squares, and hospitals; these indicators reflect the adaptability from disasters and extreme events [5,9,34].

**Table 1:** Evaluation index system of social vulnerability in Nanjing.

Variable	No.	Name	Description and Measurement	Impact	Data Source
*Demographic indicators*					[5,9]
Age structure	1	P75	Percent of population over 75 years	+	
	2	P14	Percent of population under 14 years	+	
	3	PKP	Percent of population under kindergarten and primary school age	+	
Gender	4	PFEM	Percent of females	+	
Rural	5	PAGR	Proportion of agricultural household registration	+	
Immigrant	6	PMIG	Percent of immigrants	+	
Education	7	PlowEDU	Percent of low-education population (≤9 years of education)	+	
	8	PILLITER	Percent of illiterate population	+	
Population	9	PDNSTY	Population density	+	
*Economic indicators*					[9,35,36]
Home value	10	AVEHPRI	Average house prices	-	
	11	DRET	Retail density	-	
	12	AVEHARE	Housing area per capita	-	
Construction density	13	DCORP	Corporate density	+	
	14	PCONSTR	Proportion of construction land	+	
*Facilities indicators*					[5,9,34]
Unsafe conditions	15	DKIN	Kindergarten density	+	
	16	DPRI	Primary school density	+	
	17	DMID	Middle school density	+	
	18	DNURS	Nursing home density	+	
Service facilities	19	DBUS	Bus station density	-	
	20	DPS	Park and square density	-	
	21	DHOSP	Hospital density	-	

Note: When an indicator is positively correlated with social vulnerability, the impact of this indicator is signed as “+”, indicating that the high value of this indicator may increase social vulnerability. In contrast, when an indicator has a negative relationship with social vulnerability, the impact of this indicator is signed as “-”, indicating that the high value of this indicator may decrease social vulnerability.

### 3.3. Methods

#### 3.3.1. SoVI Scores

Min–max rescaling was performed to normalise all of the indicators. A score of 0 reflects the lowest rank, and a score of 1 represents the highest. The positive index was used to standardise Formula (1), whereas the negative index was used to standardise Formula (2).

(1) $$Y_{ij}=(X_{ij}-X_{j\min})/(X_{j\max}-X_{j\min})$$

(2) $$Y_{ij}=(X_{j\max}-X_{ij})/(X_{j\max}-X_{j\min})$$

where *X_ij_*, *X_j_*_max_, *X_j_*_min_, and *Y_ij_* are the original value, maximum value, minimum value, and standardised value of the index *j* of the research unit *i*; *i* = 1, 2, ..., *m*; *j* = 1, 2, ..., *n*.

SPSS 22.0 (IBM, Chicago, IL, USA) was used for factor analysis. Principal component analysis (PCA) was used to extract principal components as new factors, which can reduce the number of variables and retain the provided main features. The maximum orthogonal rotation method was employed to minimise the multicollinearity amongst the indicators to realise the interpretation and identification of social vulnerability factors. Since those factors indeed loaded different degrees of original indicators information, the weighted method was considered for calculating SoVI scores. Information percentages loaded on these factors were used as weights. The weighted superposition calculation formula is as follows: *w_i_* is the weight of *i* factor, and *f_i_* is the *i* factor.

(3) $$\mathrm{SoVI}=\sum_{i=1}^{n}w_{i}\times f_{i}$$

To reflect spatial differences in social vulnerability, each factor and the weighted scores were classified into five levels by using ±1.0 standard deviations as standards (first decile: ‘low’, second decile: ‘medium low’, third decile: ‘medium’, fourth decile: ‘medium high’, and fifth decile: ‘high’).

#### 3.3.2. Spatial Pattern of SoVI

The *G_i_* index was used to determine whether spatial units exhibit clusters or similar phenomena throughout the study area [38]. The value range of the index is [−1, 1], where +1 indicates strong positive spatial correlation (including high or low value agglomeration), and −1 suggests strong spatial negative correlation; 0 represents no obvious spatial pattern.

The G_i_* index was used to map the spatial heterogeneity of SoVI [38]. Each analysed unit obtained a score by comparing within the context of neighbouring features. Hot spots indicates that the score of units (and its neighbours) is higher than the expected value on average, whereas cold spots indicate the opposite. The calculation formulas are as follows:

(4) $$I=\frac{n\sum {}_{i=1}^{n}\sum {}_{j=1}^{n}W_{ij}\left(x_{i}-\bar{x}\right)\left(x_{j}-\bar{x}\right)}{\sum {}_{i=1}^{n}\sum {}_{j=1}^{n}W_{ij}\sum {}_{i=1}^{n}{\left(x_{i}-\bar{x}\right)}^{2}}$$

(5) $$G_{i}^{*}=\frac{\sum_{i=1}^{n}W_{ij}x_{j}}{\sum_{j=1}^{n}x_{j}}$$

where *x_i_* and *x_j_* are the values of neighbourhoods *i* and *j*, *W_ij_* is the spatial weight matrix, and the value of *I* is between 0 and 1. G_i_* can map hot and cold spots.

#### 3.3.3. Flood Susceptibility

The ArcSWAT2012 software (USDA–ARS, Temple, TX, USA) based on the ArcGIS10.3 (Esri, Redlands, CA, USA) platform was used to simulate flood in Nanjing. DEM data, land use, soil type, and rainfall data were imported into ArcSWAT2012 for analysis. Firstly, we processed the data through ArcGIS10.3, a river grid file was converted into a vector file through the operation of ‘filled in’ (with the *Z* limit as 30 m) and ‘river network extraction’ (the confluence accumulation threshold was set as 1000) on the DEM map. Secondly, the DEM map and river vector file were imported into ArcSWAT for watershed division, and then hydrological response units were divided by importing land use, soil type, and slope data. Finally, the simulation results of runoff depth were obtained by inputting rainfall data. ArcGIS10.3 was used to overlap the runoff depth results with the neighbourhood areas of Nanjing. Nanjing was divided into 259 basins and 4754 hydrological response units. When runoff depth exceeds 0.4 m, it will cause severe urban flooding. Based on the calculated area ratios of neighbourhoods with a runoff depth exceeding 0.4 m, 99 neighbourhoods in Nanjing were divided into five levels of flood susceptibility (first decile: ‘low’, second decile: ‘medium low’, third decile: ‘medium’, fourth decile: ‘medium high’, and fifth decile: ‘high’).

## 4. Study Area and Data

### 4.1. Study Area

**Figure 2:**
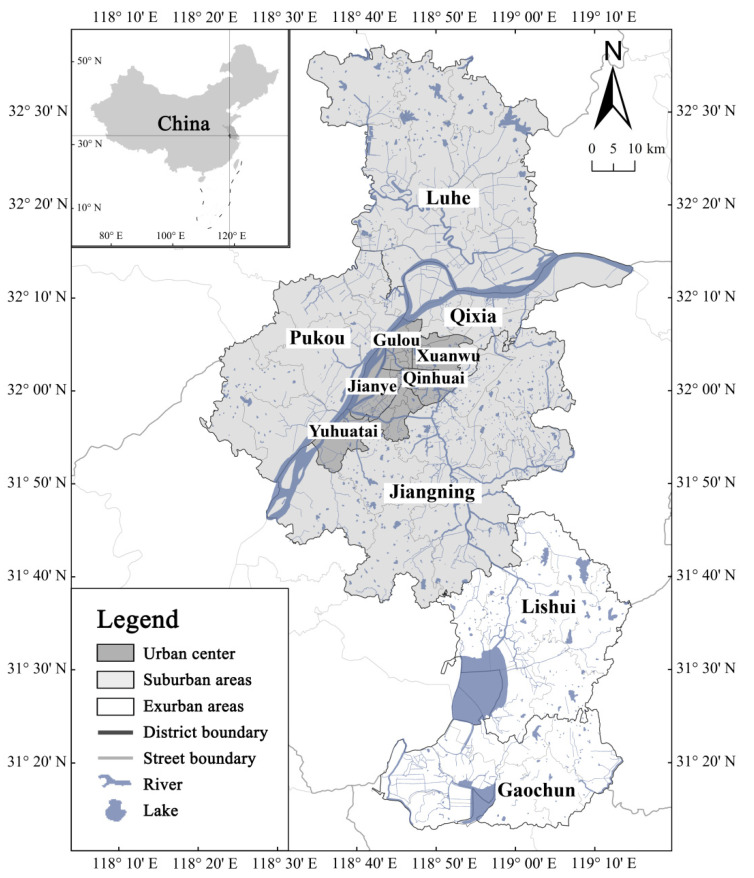
Nanjing map.

Nanjing is the capital of Jiangsu Province and the centre of the Yangtze River Economic Belt. In 2019, the land area was 6587 km^2^, and the population was 8.4 million with an urbanisation rate of 82.5% (Figure 2). The East Asian monsoon has an effect on the city, which has a humid subtropical climate. The average annual precipitation was more than 1100 mm and mainly concentrated from May to September. In 2020, Nanjing experienced the most acute flooding in summer with heavy rainstorms since 1998.

Nanjing has developed a multicentric city pattern, which represents the general model of transformation of the spatial structural of China’s large city [39]. Property-led urban growth has become the primary driver of China’s urban sprawl and renewal, including the country’s social differentiation and social exclusion. It particularly exacerbates the problems of vulnerable groups in urban areas, such as rural immigrants, the elderly, and low-income families. Public resources, such as medical care, schools, and infrastructure, are still mostly concentrated in the city centre [28,39]. Therefore, the inequality of the social space based on the differences in housing prices and public facilities increases social vulnerability. The spatial distribution in Nanjing is depicted in Figure 2. The urban centre comprises four municipal districts (Xuanwu, Qinhuai, Jianye, and Gulou), including 38 neighbourhoods. Suburban areas consist of five municipal districts (Pukou, Qixia, Jiangning, Yuhuatai, and Luhe), including 46 neighbourhoods. Exurban areas comprise Lishui and Gaochun districts, which include 15 neighbourhoods. Nanjing comprises 11 municipal districts with a total of 99 neighbourhoods.

### 4.2. Data

#### 4.2.1. Indicators of Social Vulnerability Assessment

① Demographic indicators. Data were derived from the Sixth Census of Nanjing Municipal Bureau of Statistics (2010). ② Economic indicators. Average house prices were captured from the Anjuke website (https://Nanjing.anjuke.com/, accessed on 15 December 2018), which includes sufficient resold house information in Nanjing. Data of 2,994 houses resold in 2018 were obtained by using Python and then located on the research area by Geocoding, which were used to calculate the average house price of each neighbourhood. POI data, including retail, business, and facility, came from the Baidu Map Service (http://map.baidu.com/, accessed on 21 October 2019). A total of 8658 retail businesses and 7681 enterprise companies were identified. Retail businesses were classified into eight categories according to the ‘Industrial Classification for National Economic Activities’ (GB/T 4754—2017), and the ratio of the eight industries in the retail industry was 30%, 20%, 10%, 5%, 20%, 5%, 5%, and 5% (these categories are integrated, grocery, daily necessities, cultural and sporting goods, medicine and medical equipment, power equipment, electronic product, tools, and decoration materials, respectively). ③ Facility indicators. A total of 18,333 POIs, including 152 parks, 146 nursing homes, 439 primary schools, 368 hospitals, 935 kindergartens, 192 middle schools, and 14,794 bus stops in Nanjing, were obtained. These POI data above include names, coordinates, addresses, and other relevant information.

#### 4.2.2. Flood Susceptibility Data

① Remote sensing images were obtained from Landsat 8 (NASA, Washington, DC, USA) satellite images with medium resolution (data acquisition time was September 2018). Land use data were acquired from the remote sensing images of Nanjing in 2010 in ‘GlobeLand30’. Soil type data were obtained from the publication ‘1:1 million soil type data of Jiangsu Province’. The Mercator projection correction was adopted. ② Rainfall data were obtained from Nanjing Meteorological Station. Daily rainfall data from June to September 2015 were selected to simulate the daily runoff depth under the typical flood disaster scenario in Nanjing.

## 5. Results and Discussion

### 5.1. SoVI Factors

Twenty-one social vulnerability indicators were analysed through principal component analysis. The Kaiser–Meyer–Olkin value was 0.891 (*df* = 210, *p* < 0.001), indicating that the outcome was satisfactory. According to the principle that eigenvalue > 1, four component factors were extracted. These four factors explain 75.906% of the total data (Table 2).

The first factor, representing 39.928% of the variance, was named ‘Urban Construction’. It is positively correlated with population density, corporate density, construction land proportion, kindergarten density, primary school density, middle school density, and nursing home density but negatively correlated with average house price, retail density, bus station density, park and square density, and hospital density. This factor represents the facility construction situation and economic level, which comprehensively reflects the development status of each neighbourhood. The second factor was labelled ‘Vulnerable Groups’. It has five main variables and explains 18.200% of the variance; amongst them, only the housing area per capita represents house condition loads negatively. The variables of elderly people, agricultural households, low education, and illiterate population loading positively show that this factor is related to groups that are vulnerable to disasters and are difficult or take a long time to recover. The third factor was named ‘Children’. It represents 11.306% of the variance, and its two positive variables are the percentage of population under 14 years and under kindergarten and primary school age. The fourth and last factor has two variables and represents 6.472% of the variance. It was named ‘Female and Migrant’ because its variables are the percentages of females (loading negatively) and immigrants (loading positively). These two groups are likely treated unequally and are at disadvantages in urban resource allocation and career selection.

**Figure 3:**
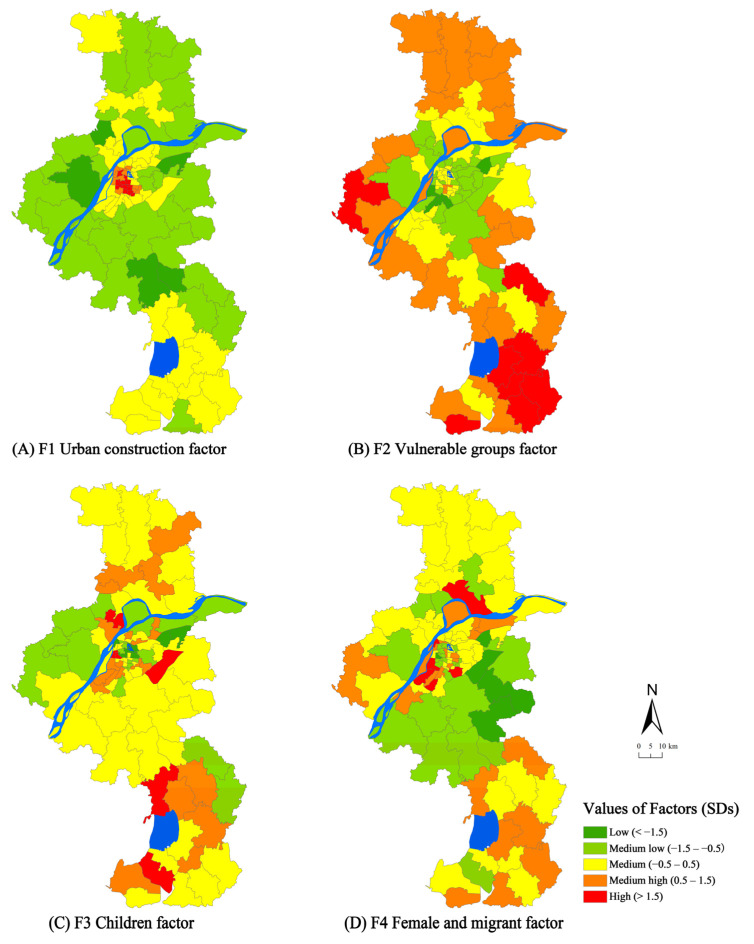
The spatial pattern of all factors of social vulnerability in Nanjing.

We analysed and interpreted the four factors to discover the spatial pattern of social vulnerability (Figure 3). The Urban Construction factor (Factor 1) was low in suburban areas but high in the urban centre. Public facilities and infrastructures tend to be high around the urban centre and low in exurban areas. When flood occurs, high-density construction areas and low-lying communities face substantial risk of loss [15]. The mid-value for exurban areas shows a lack of public facilities and a low risk of flood losses. Suburban areas have low-range scores. These areas also have sufficient public facilities and infrastructures, including good accessibility, compared with exurban areas, thus reflecting the relative correlation of the construction density and social vulnerability.

The Vulnerable Groups factor (Factor 2) scored low in the urban centre and high in exurban areas, exhibiting a central–peripheral pattern in general (Figure 3). The elderly and people with low education are distributed in exurban areas. These areas have higher social vulnerability than the urban centre and suburban areas. The rapid increase in the cost of urban housing has made it difficult for vulnerable groups, such as low-income individuals, to buy homes in the city centre; renters are also more vulnerable than homeowners to flood [40]. The enlarged gap in housing prices pushed vulnerable groups to live in exurban areas [39]. The older populations are more vulnerable than others due to mobility and health issues [40]. Low and medium scores were recorded in urban and suburban areas, respectively; residents in these areas have a relatively high income and education level, making them more conscious of flood risk than others.

High values of Children factor (Factor 3) were scattered throughout suburban and exurban areas. These areas are mainly located in new towns and have high-level real estate development; as a result, increasing numbers of kindergarten and primary schools have been built. Children are more vulnerable to flood due to their lack of self-protection capacity [5,27,41]. Low values were found in the urban centre, which has high-quality public facilities and support services, such as renowned public schools and famous hospitals. High housing prices push many parents and children to become commuters. The misallocation of education resources restricts social mobility and further exacerbates urban socio-spatial differentiation [42].

The Female and Migrant factor (Factor 4) scored high in the urban centre and exurban areas but low in suburban areas (Figure 3). Rural migrants are located in the urban centre and exurban areas, which usually have limited social networks and tend to be ignored during flood periods [40]. The urban centre provides many job opportunities to migrants, whereas exurban areas have low house rents to save on living costs. In addition, exurban areas have many labour-intensive industries, such as the manufacturing industry, which can attract migrants to live and work. Rural migrants tend to concentrate in areas with large job opportunities and low living costs. Females have lower incomes and fewer financial resources than males, which makes females vulnerable to flood [27,40,41,43].

### 5.2. SoVI Index

**Figure 4:**
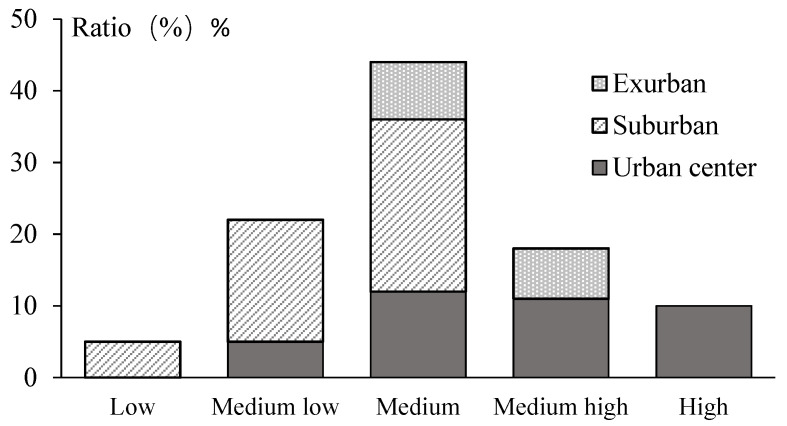
The proportion of SoVI scores in Nanjing.

Scores of all these factors were summed according to the weighted superposition method, which resulted in SoVI scores (Figure 4). SoVI ranked high in 10 neighbourhoods (10.1%), which are located in the urban centre. It was low in five neighbourhoods (5.05%), which are found in suburban areas. SoVI ranked medium in 44 neighbourhoods (44.44%), which were scattered throughout the whole city. It ranked medium low in 22 neighbourhoods (22.22%), which are located in the urban centre and suburban areas. SoVI was medium high in 18 neighbourhoods (18.18%), which were found in the urban centre and exurban areas.

**Figure 5:**
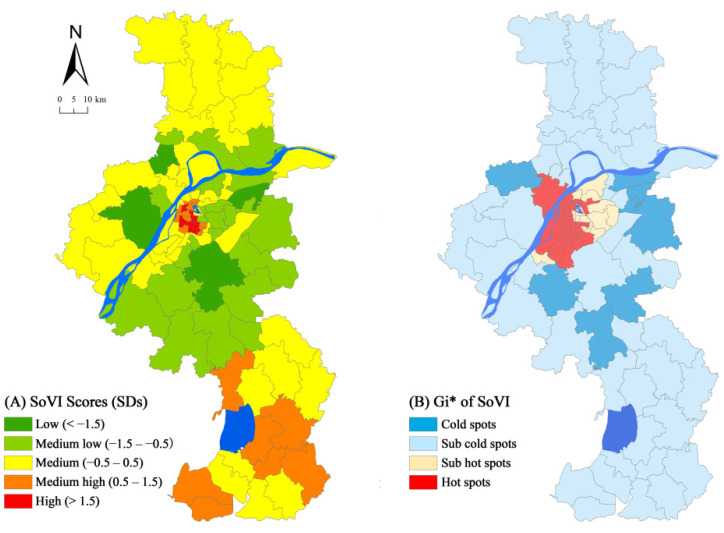
Social vulnerability map (**A**) and hot spot analysis (**B**).

High scores of SoVI were distributed within the urban centre, especially in the inner city (Figure 5A). People who live in the urban centre, particularly those who dwell in old houses without elevators and property management, are at risk of urban floods. The density of vulnerable people, such as the elderly, females, and rural migrants, has a significant impact on exposure. These people prefer to live in the urban centre with adequate public infrastructures and facilities (Xinjiekou, Hunanlu). Medium high scores of SoVI were distributed within the urban centre and exurban areas (Figure 5A). Although the density of the elderly in exurban areas was lower than in the urban centre, those of children and females were still high, increasing the proportion of migrants in exurban areas (Jingqiao, Shiqiu, Zhuanqiang). Low scores of SoVI were distributed within suburban areas. These areas have enclosed communities and luxurious villas and high green plot ratios; these factors decrease the risk of social vulnerability (Xianlin, Xigang).

The Global Moran’s I was 0.466, the standardised *Z* value was 19.253, the critical value of significance level greater than 1% was 2.58, *p* < 0.01, all of which indicate that SoVI has a positive significant spatial autocorrelation. Local Gi* displays a central–peripheral pattern in social vulnerability (Figure 5B). The urban centre was inhabited by 48 neighbourhoods, with hot spots (34.34%) and sub-hot spots (14.14%). Cold spots (7.07%) and sub-cold spots (44.44%) were situated sparsely in suburban and exurban areas, including 51 neighbourhoods. At the community level in Nanjing, SoVI revealed a central–peripheral pattern.

The Gi* of SoVI highlighted the agglomeration features in the urban centre; those in the vulnerable groups, such as children, the elderly, and rural immigrants in the urban centre, prefer to live in places with convenient public facilities and efficient schools and hospitals. Reversely, given that the densities of public facilities and infrastructures in suburban and exurban areas are low, the potential loss of flood is modest. As a result, it has a low value of social vulnerability. To put it in another way, the spatial heterogeneity of SoVI reflects social–spatial inequality. It can be defined as the disparities across cities in terms of economic, environmental, and social differentiation. The mismatch of public resource supply and demand aggravates social differentiation under the market-oriented reform.

The distribution patterns of social vulnerability have been discussed at different spatial scales, such as national, regional, and local [20,44,45]. SoVI levels were high in the city centre and low in the exurban areas of Great Lisbon [17]. For the city of Rotterdam in the Netherlands, SoVI values were high in downtown and low in the suburbs [4]. The central–peripheral pattern of SoVI can also be found in Beijing, Shanghai, and Shenzhen [23,27,28]. In comparison, the spatial pattern of Nanjing exhibited high values in the urban centre and low values in suburban and exurban areas. Nanjing has been experiencing rapid economic growth and urbanisation. Dramatic land use changes are associated with the rapid expansion of impervious surface. For example, Hexi New Town in Jianye District suffers severe flooding during rainy seasons. Social differentiation and social vulnerability are rapidly overlapping in terms of spatial heterogeneity [39].

### 5.3. Flood Susceptibility

**Figure 6:**
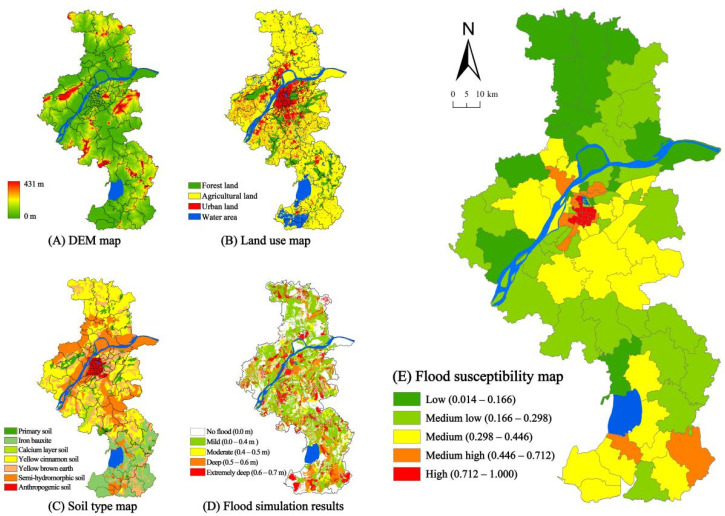
Simulation process of flood susceptibility.

The spatial distribution of urban flood risk shows that low, medium low, medium, medium high, and high are applicable to 17 (17.17%), 27 (27.27%), 26 (26.26%), 14 (14.14%), and 15 (15.15%), respectively (Figure 6). Almost all areas with high and medium-high flood risks in cities are located in the urban centre and exurban areas. Most suburban and exurban areas are at low and medium-low flood risks. Flood susceptibility in Nanjing is mostly determined by the elevation and green space ratio. Some areas adjacent to the Yangtze River are prone to water accumulation due to low-lying terrain [16]. The rapid acceleration of urbanisation has caused the surface of the natural ecosystem to become impermeable, and most rainfalls have been transformed into surface runoffs. Therefore, the flood susceptibility in Nanjing is influenced by the interaction of coupled human–environmental systems.

### 5.4. Combining Flood Susceptibility and Social Vulnerability

**Figure 7:**
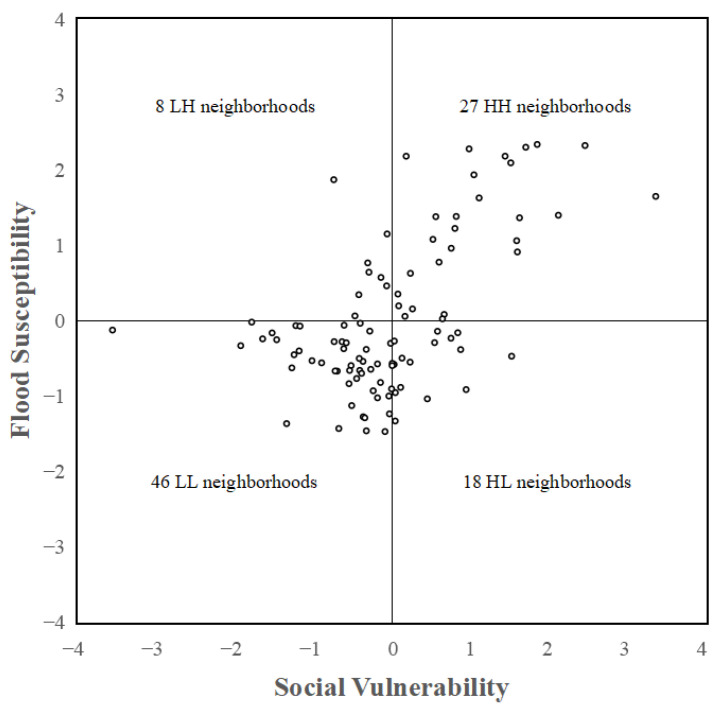
A two-dimensional coordinate system for social vulnerability and flood susceptibility.

**Figure 8:**
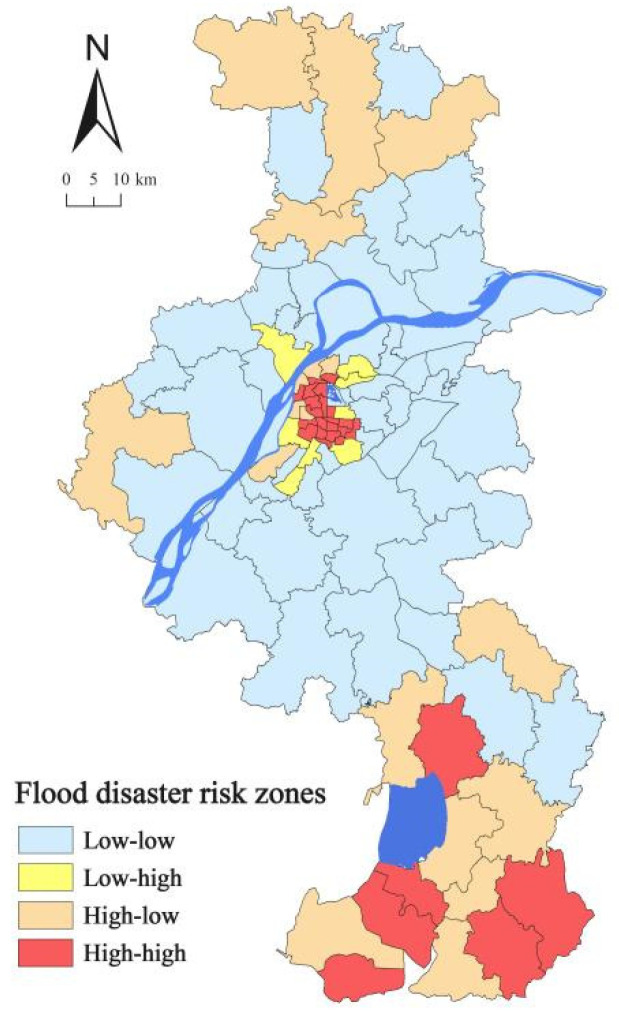
Flood risk zones in Nanjing.

By analysing social vulnerability and flood susceptibility, the two are plotted in a 2D coordinate system. The result of person correlation is 0.560, indicating that social vulnerability is positively correlated with flood susceptibility. Most neighbourhoods are centralised in the L–L range (45.46%), then in the H–H range (28.28%). Only a few neighbourhoods emerge in the L–H range (8.08%) and H–L range (18.18%) (Figure 7). Four categories have been identified to explore the correlation between social vulnerability and flood susceptibility:

The first category is L–L range, which have low social vulnerability and low flood susceptibility. Figure 8 shows that 45.46% of the neighbourhoods, the majority of which are in suburban areas, have low social vulnerability and flood susceptibility. With the rapid urbanisation, an increasing number of residential communities were built in the suburban areas. However, the facilities of education and public services lag behind the residential communities, the density of population is also lower than that of the urban centre, and people usually have strong risk perceptions to adapt flood risk management [28,39]. These neighbourhoods have relatively low flood susceptibility and can adapt to urban flooding.

The second category is H–H range, which have high social vulnerability and high flood susceptibility. The majority of neighbourhoods in the inner city and exurban areas have high social vulnerability and flood susceptibility, accounting for 28.28% of all neighbourhoods (Figure 8). Nanjing is located in low mountains, hills, and plains, which causes low-lying areas in the urban centre to be prone to waterlogging [16]. Two big lakes, Shijiu and Gucheng Lakes, are located in the exurban areas, which are the reasons for the potential flood risk. Moreover, most of the vulnerable people are scattered in the exurban areas, which increases the level of social vulnerability. Population density and public facilities are high in the H–H range. When a flood occurs, the inner city and exurban areas are at a significant risk of flooding. People’s risk awareness and emergency education should be raised, and public service delivery to the suburbs should be improved.

The third category is H–L range, which have high social vulnerability and low flood susceptibility. Approximately 18.18% of the communities have high social vulnerability and low sensitivity to floods, most of which are in exurban and suburban areas (Figure 8). In suburban areas, the risk of flood is low, and the level of social vulnerability is high; female, rural migrants, elders, and low educated people reside in suburban and exurban areas [28,39]. In the H–L range, people are generally not at risk of flooding because of the low probability of flood events [15], Meanwhile, the H–L range is still vulnerable and likely to be disproportionately affected [41]. Compared with the urban centre, suburban and exurban areas face more difficult challenges, including vulnerable populations (rural immigrants, females, and children) and lack of public services. The government should balance public resources, such as medical resources, prestigious schools, and convenient public transportation.

The last category is L–H range, which have low social vulnerability and high flood susceptibility. Of the 8.08% of neighbourhoods that have low social vulnerability and high flood susceptibility, most are located in the urban centre L–H range (Figure 8). These areas usually have a high level of economy, abundant public facilities, and high-income and highly educated people. Some rapidly urbanising neighbourhoods, such as the Xinlong neighbourhood in Hexi New Town, which is located in low-lying areas along the Yangtze River, have experienced severe flooding [16]. Nevertheless, people in the Xinlong neighbourhood have high income and high education. They have relatively low social vulnerability. Urban expansion must consider flooding when selecting new city sites, such as low-lying areas and those close to large waters. Drainage facilities require advanced planning and long-term maintenance to mitigate the risk of flooding [46].

## 6. Conclusions

This study takes Nanjing as an example of overlapping social vulnerability and flood susceptibility. The findings reveal that social vulnerability to floods in Nanjing follows a central–peripheral pattern. Specific implementations of fast-growing cities with high risks of urban floods in developing countries are provided.

This research indicates the importance of combining social vulnerability and flood susceptibility into a unified place-based framework. The social vulnerability pattern varies from the urban centre to exurban areas, showing a central–peripheral pattern. In the urban centre and exurban areas, high and medium-high social vulnerability are observed, whereas low social vulnerability is discovered in suburban areas. Considering that Nanjing is a city prone to urban flooding, we combine social vulnerability and flood susceptibility to identify the vulnerable places that can be classified into four categories. The H–H range of social vulnerability and flood susceptibility is concentrated in the inner city of the urban centre because of the high density of buildings and urban structure factor. Exurban areas also have the H–H range of social vulnerability and flood susceptibility due to poor urban construction and vulnerable population. Meanwhile, the factor that contributes to the H–L range is mainly located in suburban and exurban areas. These places are inhabited by a vulnerable population and lack of public facilities. The factor that contributes to the L–H range is mainly located in the urban centre. These areas are undergoing rapid urbanisation and have a dense population.

Previous studies on flood risk assessment mainly emphasised physical vulnerability, ignoring social vulnerability to risks and spatial changes in these dimensions [17,47,48,49,50]. Rapid urbanisation and marketisation have increased the risk of flooding by overlapping spatial heterogeneity and social differentiation. The spatial pattern of social vulnerability can be found in comparable forms all over the world, such as Sao Paulo, Dhaka, Beijing, and Shanghai [23,27,51,52]. The enlarged gap in housing prices, resident incomes, and public facilities has been widening in Nanjing. In a highly marketised system, scarce resources can help reshape geography.

Our findings have various policy implications for reducing social vulnerability. Firstly, flood hazard maps should be established as an important tool to help China manage its flood risk. The government should publish risk maps to identify the main flood risk areas. These flood risk maps can be used to identify potentially vulnerable areas and assess what type of dangerous process affects the vulnerable population [17,53]. Publishing the range of cumulative rainfall, especially social media data (e.g., Twitter and Flickr), is insufficient; generated texts and photos can also be collected to generate real-time flood risk maps [46].

Secondly, public resource allocation should be planned and constructed in exurban areas. Most public resources are still mainly concentrated in the urban centre, such as medical care, infrastructure, and schools. The unbalanced distribution of public resources leads to continuous escalation in spatial heterogeneity and social vulnerability. As shown in this study, excessive public resources are located in the urban centre; attracting high-density populations increases the risk of flooding. People in the urban centre tend to live in welfare public housing, which was built in the 1980s and 1990s and lacks modern amenities and services, such as parking spaces, elevators, and property management [39]. Previous studies revealed that high-density neighbourhoods are vulnerable to catastrophic property losses [54]. The risk of urban flooding in exurban areas is also high. The high densities of children and females in exurban areas increase social vulnerability. The government should intervene by providing public resources to exurban areas to achieve spatial balance.

Thirdly, the adoption and instalment of flood insurance are important in communities and residents to alleviate property losses. Based on past experiences of negative effects on neighbourhood property values, such as houses and vehicles, the government should formulate flood insurance policies to minimise the interference of urban floods in people’s daily lives. Past research argued that properties located in flood-prone areas can degrade in value, lowering the total community housing prices and even causing population displacement. In addition, the ability to precisely estimate populations potentially affected by flood hazard, particularly with regard to vulnerable subgroups, can improve community resilience, flood hazard planning, and city inclusiveness [55,56]. Last but not least, high-quality drainage infrastructure should be installed to prepare for regular urban flooding.

Our findings demonstrate patterns of social vulnerability to flood in Nanjing on the basis of an analysis of social vulnerability and flood susceptibility. Considering the data availability, this study is mostly based on the 2010 census data; the 2020 census data should be obtained to compare changes in social vulnerability. In the future, potential vulnerable regions should be highlighted, and the ability to be more resilient than before should be prioritised.

## Figures and Tables

**Table 2:** Results of PCA: the components, main variables, and their interpretable variances.

Component Factors	No.	Name	Loading	Eigenvalue	Interpretable Variance	Cumulative Variance Contribution Rate (%)
Factor 1: Urban construction	9	PDNSTY	0.925	10.593	39.928	39.928
	10	AVEHPRI	−0.596			
	11	DRET	−0.874			
	13	DCORP	0.827			
	14	PCONSTR	0.818			
	15	DKIN	0.875			
	16	DPRI	0.912			
	17	DMID	0.647			
	18	DNURS	0.610			
	19	DBUS	−0.846			
	20	DPS	−0.586			
	21	DHOSP	−0.768			
Factor 2: Vulnerable groups	1	P75	0.863	2.531	18.200	58.128
	5	PAGR	0.729			
	7	PlowEDU	0.737			
	8	PILLITER	0.695			
	12	AVEHARE	−0.588			
Factor 3: Children	2	P14	0.892	1.562	11.306	69.434
	3	PKP	0.922			
Factor 4: Female and Migrants	4	PFEM	−0.704	1.253	6.472	75.906
	6	PMIG	0.834			
